# Ebola virus disease sequelae and viral persistence in animal models: Implications for the future

**DOI:** 10.1371/journal.ppat.1012065

**Published:** 2024-03-21

**Authors:** Olivia Durant, Andrea Marzi

**Affiliations:** Laboratory of Virology, Division of Intramural Research, National Institute of Allergy and Infectious Diseases, National Institutes of Health, Hamilton, Montana, United States of America; University of Arizona, UNITED STATES

## Abstract

Ebola virus disease (EVD), caused by infection with Ebola virus, results in severe, acute illness with a high mortality rate. As the incidence of outbreaks of EVD increases and with the development and approval of medical countermeasures (MCMs) against the acute disease, late phases of EVD, including sequelae, recrudescence, and viral persistence, are occuring more frequently and are now a focus of ongoing research. Existing animal disease models recapitulate acute EVD but are not suitable to investigate the mechanisms of these late disease phenomena. Although there are challenges in establishing such a late disease model, the filovirus research community has begun to call for the development of an EBOV persistence model to address late disease concerns. Ultimately, this will aid the development of MCMs against late disease and benefit survivors of future EVD and filovirus outbreaks.

## What are Ebola virus and Ebola virus disease?

Since it was first identified in 1976, Ebola virus (EBOV) of the *Filoviridae* family has been a cause for public health concerns in Africa. Ebola virus disease (EVD), caused by infection with EBOV, is known as a severe, acute illness resulting in a high mortality rate due to multisystem organ failure and hypovolemic shock [1]. Acute disease symptoms include fever, fatigue, muscle pain, vomiting, diarrhea, rash, and internal and external bleeding with symptoms typically occurring 2 to 21 days postinfection [2]. During the 2013 to 2016 West African EVD epidemic, the severity and mortality of EVD became evident on a large scale, with more than 28,000 people infected and over 11,000 dead [1].

## Why should we investigate EVD sequelae, recrudescence, and EBOV persistence?

The unprecedented scale of the 2013 to 2016 West African EBOV epidemic led to more EVD survivors than there had ever been previously, which has enabled medical and scientific communities to observe a new, poorly understood late phase of the illness during and after convalescence: Some EVD survivors exhibit disease sequelae and experience symptoms of recrudescence [3]. Sequelae, defined as a condition resulting from a prior disease [4], has been noted following infection with many viruses including several different ebolaviruses (Ebola, Sudan (SUDV), Bundibugyo and Taï Forest virus) and occurs after recovery from acute illness [3].

Among other symptoms, EVD sequelae, which are not necessarily related to viral persistence, include ocular issues, most commonly uveitis, alopecia, anorexia, arthralgia, various skin disorders, and neurological symptoms, including encephalopathy, meningitis, and hearing loss. It has been postulated that the appearance of sequelae may be due to the persistence of infectious EBOV in which the virus remains “hidden” (e.g., undetected by the immune system) in immune-privileged sites [3]. Indeed, data from a 48-month study which followed a cohort of 802 EVD survivors in Guinea demonstrated a prevalence of neurologic sequelae at 30.55%, musculoskeletal sequelae at 5.80%, and ocular sequelae at 4.24% [5]. Duration of sequelae varied with the highest mean duration of 2013 days reported for neurologic sequelae. The authors suggest that the development of sequelae may be impacted by the acute disease course [5].

Distinct from EVD sequelae, EVD recrudescence is the reoccurrence of EVD symptoms, and potential infectious virus, after apparent clinical recovery from the illness. This has been described during several EBOV and MARV outbreaks and reached the spotlight during the West African EBOV epidemic. It was also highlighted more recently during the 2018 to 2020 outbreak in the Democratic Republic of Congo [3,6–8]. During the West African EBOV epidemic, EBOV RNA was found to be harbored in clinically recovered individuals in immunologically privileged sites, defined as comparments or tissues of the body in which there is no immunological response [9], including parts of the eye, products of conception (placenta and amniotic fluid), breast milk, urine, sweat, and semen. Several chains of transmission were linked to sexual transmission during the epidemic [3].

Taken together, the clinical burden of EBOV persistence in immunologically privileged tissues and the subsequent risk of transmission, as well as the development of sequelae and recrudescence, are cause for public health concerns and, therefore, are a priority focus of the EBOV research community. Additionally, MARV and SUDV may also be sexually transmitted and may persist in immunologically sequestered sites [8,10–12]. Given the recent outbreaks of MARV and SUDV, there is a need for continued research efforts on sequelae, persistence, and recrudescence for all filoviruses. It is crucial to gain a detailed understanding of the relationship between filoviral persistence and recurrence of acute filovirus disease (FVD) as the global public health implications and potential strain on local health systems by FVD outbreaks possibly started by filovrius recrudescence are enormous. These research findings will benefit survivors of recent filovirus outbreaks and survivors of inevitable future outbreaks.

## What is known about EVD sequelae and EBOV persistence in animal models?

EBOV animal disease models were developed to cause severe disease and lethality to set a high bar for medical countermeasure (MCM) efficacy evaluation during acute disease. Due to the severity of disease after EBOV infection in these animal models, including nonhuman primates (NHPs), rodents, and ferrets, most animals reach endpoint criteria and are euthanized, leaving little room to evaluate disease sequelae, viral persistence, or recrudescence [13,14]. To date, EBOV research in animal models has predominantly focused on the investigation of the disease course and the development of MCMs, including preclinical testing of vaccines and treatments, such as monoclonal antibodies (mAbs).

EVD sequelae and EBOV persistence have rarely been studied as primary study endpoints during live animal studies but have been investigated retrospectively and tangentially in surviving animals from MCM studies and pathogenesis/natural history studies. Examples are retrospective analyses of archived rhesus and cynomolgus macaque tissues for EBOV persistence by Zeng and colleagues [14] and of archived cynomolgus macaque tissue for MARV persistence by Coffin and colleagues [8]. A notable exception to this trend to investigate tissue samples retrospectively is a study by Clancy and colleagues that specifically investigated EBOV persistence in a mouse model [15].

**Fig 1:**
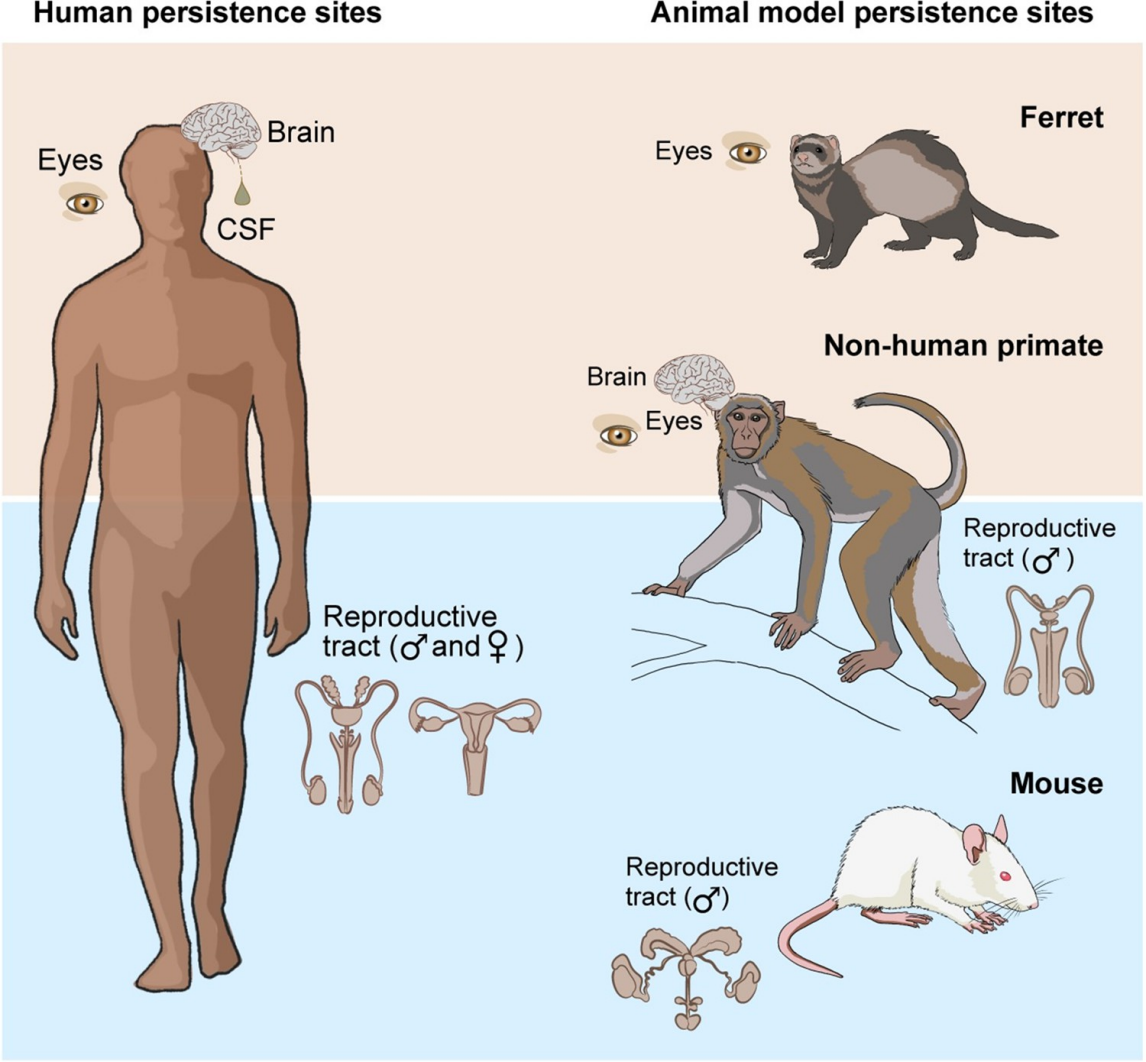
EVD persistence and sequelae in humans and animal models. Illustration of the affected body parts in humans and animal models by EBOV persistence and sequelae. CSF, cervical spinal fluid.

A summary of studies investigating filovirus sequelae and viral persistence in animal models can be found in Table 1. Of note, the eye, various brain structures, and male reproductive tract, specifically the epididymis, of various animal models have been found to exhibit signs of EBOV persistence by detection of viral RNA or antigen [14–19] (Fig 1). This phenomenon appears to mirror EBOV persistence in humans as EBOV has been found in humans in the aqueous humor, conjunctival swabs, cerebrospinal fluid, semen, and other body compartments [3], suggesting that these animal models may be appropriate to study EBOV persistence. Several animal studies detail EVD neurologic sequelae [16,17,19–21] adding to the relevance of these animal models in sequelae and persistence research.

**Table 1:** Summary of filovirus sequelae and persistence in animal models.

Animal model		Agent	Survival	MCM	EVD sequelae	EBOV RNA or antigen detection	Impacted anatomical and cellular structures
Mouse	Male CC011 [10]	MA-EBOV	90%	None	N/A	Epididymal epithelial cells by IHC (50%); epididymis epithelial cells at 35 DPI (100%)	Mild spermatogonia degeneration (20%), interstitial epididymal inflammation (50%), testicular immunoreactivity (20%), pampiniform vasculitis (100%)
	Male CC021 [10]	MA-EBOV	90%	None	N/A	Epididymal epithelial cells by IHC (50%)	Epididymis (40%), mild spermatogonia degeneration (10%), interstitial epididymal inflammation (50%), testicular immunoreactivity (10%), pampiniform vasculitis (75%)
	Female *IFNAR* ^*-/-*^ [10]	MA-EBOV	0% IP, 20% IVa	None	N/A	N/A	Diffuse macrophage infiltration of the myometrium
	Female *IFNAR* ^*-/-*^ [10]	EBOV-Makona	0% IP, 20% IVa	None	N/A	N/A	Diffuse macrophage infiltration of the myometrium
NHP	Rhesus Macaque [11]	EBOV-Kikwit	N/A (retrospective tissue analysis, *n* = 36)	Monoclonal antibodies	Neurological relapse	Brain ventricular system (19.4%)	Chronic inflammation of ventricular system (ventriculitis); chordoid plexus vasculature; CD68+ T cells, meningoencephalitis (42%); near complete loss of CSF-secreting ependymal cells; disruption of blood-CSF barrier
	Rhesus macaque [11]	EBOV-Kikwit	N/A (retrospective tissue analysis, *n* = 15)	None	None	None	Ocular inflammation (27%)
	Rhesus macaque [9]	EBOV-Kikwit	N/A (retrospective tissue analysis, *n* = 112; survivor *n* = 8)	Non-vaccine MCM or untreated	N/A	Overall: ocular (11.54%, 9/78); testes (1.32%, 1/76); brain (1.25%, 1/80) Untreated survivors (*n* = 8): uveitis (75%, 6/8); brain (12.5%, 1/8); epididymis (12.5%, 1/8)	Eye, testes, brain, epididymis, CD68+ T cells
	Rhesus macaque [15]	EBOV-Makona	N/A (one NHP from a 15-NHP study)	rVSV-EBOV vaccine	Facial and scrotal edema, tachypnea, neurological (ataxia, head and intention tremors, dysmetria), uveitis, acute encephalitis	Lung, seminiferous tubules, epididymis, eye (retinal pigmented epithelium and sclera), brain (cerebral cortex, meninges)	Lung, brain, reproductive tract
	Rhesus macaque [12]	EBOV-Makona	N/A (one NHP from a 15-NHP study)	Monoclonal antibody cocktail (3 formulations; FVM04 and CA45 in affected NHP)	Delay of severe EVD associated with mutation in viral genome (E545D in EBOV GP)	Brain stem, stomach, mesenteric lymph node	Brain stem, stomach, mesenteric lymph node
	Rhesus macaque [13]	EBOV-Kikwit	N/A (a case study of an untreated female survivor)	None	Swelling of left upper eyelid, conjunctiva, delayed clinical decline, choriomeningoencephalitis	Left eye (sclera, peri-optic neuritis), brain (choroid plexus), stomach, proximal duodenum, pancreas	Eye (sclera), brain, digestive tract, pancreas
	Rhesus macaque [14]	EBOV-Kikwit	*n* = 6, 0% (animals had protracted disease course and atypical pathological findings)	Recombinant nematode anticoagulant, recombinant human activated protein C, human monoclonal antibody KZ52, recombinant human interferon-beta1a	Neurological symptoms for animals surviving past 20 days (hind limb paralysis, tremors), choriomeningoencephalitis, ocular inflammation, candidiasis	Brain (neuropil), eye (cornea, retina, and/or pia arachnoid of the optic nerve), pancreas, lung, thyroid (follicular or parafollicular)	Pancreas, lungs, brain, eye, thyroid
	Rhesus macaque [16]	EBOV-Kikwit	N/A (single animal with an abnormal disease course)	Replication-defective recombinant human adenovirus serotype 5 expressing interferon, ZMAb	Neurological (paralysis, headache (holding head in paws))	Cerebrospinal fluid	Nervous system, cerebrospinal fluid, suspected macrophage involvement to cross the blood–brain barrier
	Cynomolgus macaque [9]	EBOV-Kikwit	N/A (retrospective tissue analysis, *n* = 48)	Vaccine candidates	N/A	None	None
	Cynomolgus macaque [7]	MARV-Musoke	N/A (retrospective tissue analysis, *n* = 97)	Antiviral treatment	N/A	Inflammatory cells of the ciliary process (4.9% 3/61), Sertoli cells (30.1%, 22/73)	Eye, reproductive tract
Other	Ferret [18]	EBOV-Kikwit	0%, *n* = 20	None	N/A	None had antigen or RNA in any tissue at the end of the 14-day study. However, RNA was detected in immune-privileged sites including the eye, lung, reproductive tract, and blood vessels at endpoint.	Eye, lung, reproductive tract

CSF, cervical spinal fluid; DPI, days postinfection; EBOV, Ebola virus; IHC, immunohistochemistry; IP, intraperitoneal; Iva, intravaginal; MA-EBOV, mouse-adapted EBOV; MARV, Marburg virus; MCM, medical countermeasure; N/A, not applicable.

## What is the significance of EBOV persistence in animal models?

While EVD sequelae would burden local healthcare systems with disease management, sequelae themselves are not transmissible. Viral persistence, however, is of major concern for the potential to reignite disease outbreaks, which would further stress health care systems and potentially impact many people, thus making it a research priority. Social stigma is also a concern for survivors of EVD and those experiencing EVD sequelae [3]. This holds true in cases of uveitits even if these survivors test negative for EBOV in conjunctivae and tears [22].

Although persistence and sequelae studies uniformly found that organ targets of acute EVD (liver, spleen, and lymph nodes) were not sites of significant viral RNA persistence, clearance of EBOV from the blood and these target tissues does not mean EBOV is cleared from other organs or tissues [14,16,17,20]. Several studies on EBOV persistence note that some NHPs that received MCMs, specifically mAbs, were able to initially survive levels of viremia that likely would have otherwise been fatal [14,16,17,20]. Subsequently, these NHPs exhibited viral persistence, suggesting that high levels of viremia may seed EBOV into immunologically privileged sites leading to viral persistence, and potentially recrudescence and sequelae. In some cases, EBOV persistence and sequelae in animal models, and even humans, may hinge upon a therapeutic-dependent persistence mechanism [14,16,23] which is a risk we take with many viral infections for which we have approved treatments. The best example for this phenomenon is human immunodeficiency virus (HIV) for which many tretaments exist with known resistance mutations [24–26]. It was hypothesized that a mutation in the EBOV GP, specifically E545D [17], may be correlated with viral persistence, though other, currently unknown mutations may also be associated with viral persistence.

It was also hypothesized that blood vessels may play a key role in disseminating EBOV to immune-privileged sites, but such a mechanism remains poorly understood [14,27]. CD68+ macrophages/monocytes have been found to harbor EBOV in persistently infected tissues [8,14,16]. Liu and colleagues postulate that EBOV persistence in the brain’s ventricular system arises from the choroid plexus vasculature, with CD68+ T cell macrophages as the site of viral persistence [16]. Much remains to be understood about the mechanisms of establishing EBOV persistence in immunologically privileged sites and if and how EBOV may be “re-activated” to instill a productive infection.

## Why do we need an animal model of EBOV persistence?

Given that the incidence of EVD and other filovirus outbreaks has been increasing in recent years—a trend likely to continue—studying all facets of disease is necessary. As MCMs have been developed and lead to increased survival, it is likely that a greater incidence of sequelae will be observed.

The PREVAIL III study has documented the most frequent EVD sequelae among a cohort of EVD survivors in Liberia. Results of this study show evidence for intermittent EBOV RNA positivity in semen and suggest that a higher viral load during acute illness may be associated with this persistence [28]. This persistence has the potential to start new outbreaks of EVD. Given that persistence and sequelae are well documented and are known to cause a burden to health care systems, investigating MCMs for all phases of EVD is important to pursue. It is also imperative to understand the mechanisms of EBOV persistence and recrudescence in order to develop MCMs against these phenomena. Additionally, education of the public about this risk and possible measures of prevention (e.g., the use of condoms) should be included in future campaigns.

The development of an animal model of EBOV persistence would allow researchers to study sequelae, viral persistence, and recrudescence, and enable the development of MCMs for these stages of illness. Although development of such a model would likely be difficult for several reasons, there are several options under exploration and in need of further development.

## What should be considered when developing an animal model for EBOV persistence and sequelae?

One hurdle to consider when developing an animal model for EBOV persistence and EVD sequelae is time. Humans have been known to exhibit sequelae beginning months after recovery from acute EVD and can show EBOV RNA persistence for months to years [28]. Most high containment animal research facilities cannot house EBOV-infected and surviving animals for that long. To study sequelae, persistence, and recrudescence, animals need to survive long enough for these processes to occur. Regardless of animal size, conducting a long-term animal study (at least 40 days after infection) is resource intensive but could prove useful. Another hurdle is the apparent randomness of EBOV persistence and EVD recrudescence. Although NHPs are the gold standard for recapitulating EVD, we cannot predict which animal might or might not be affected or how many out of a study cohort will develop sequalae or recrudescence. Proper investigation would require very large group sizes in order to capture enough observations to decipher significant events.

Although several animal models have been explored and considered, none is perfect. NHPs are a poor persistence model to examine the nature of sequelae and persistence because wild-type (WT) EBOV is >90% lethal in NHPs [13]. An MCM-persistence model may be a viable option, but NHPs are very resource intensive to maintain. Mice have been the only animal model explicitly investigated as a persistence model and should be further explored [15]. A draw back, however, is that mouse-adapted EBOV must be used because immunocompetent mice do not exhibit disease after WT-EBOV infection [13]. Although a persistence model may or may not be needed in order to study EVD sequelae in an animal model, linking the development of such models may prove useful for downstream applications including MCM development.

Ferrets are currently being investigated as an animal model for various WT ebolaviruses and have been found to display signs of disease after intramuscular as well as mucosal infection with several of these viruses [27]. This model deserves further exploration regarding its suitability as a model of viral persistence and recrudescence through the analysis of immunologically sequestered sites such as the eye, brain, and reproductive tract.

Among authors that have published work about EBOV persistence and EVD sequelae in animal models, there is a resounding call for the development of an EBOV animal persistence model, which demonstrates that this community is actively interested in the further study of late disease. The development of such a model would enable researchers to study mechanisms of persistence, recrudescence, and sequelae, and, ultimately, develop MCMs to treat disease sequelae or inhibit their appearance. As it is likely that there will be many more cases of human disease after filovirus infection, survivors in the coming years would greatly benefit from the development of such MCMs.

Finally, and in light of the number of EVD survivors with the recent outbreaks, human studies investigating immune responses over a prolonged period of time after acute EVD should be possible and encouraged. The PREVAIL III study [19] is an example of what can be achieved and we should build upon this data. Such studies likely present our best opportunity to gain additional insight into EVD sequelae, EBOV persistence, and recrudescence.
